# Zeta Inhibitory Peptide as a Novel Therapy to Control Chronic Visceral Hypersensitivity in a Rat Model

**DOI:** 10.1371/journal.pone.0163324

**Published:** 2016-10-24

**Authors:** Ying Tang, Aiqin Chen, Yu Chen, Lixia Guo, Hengfen Dai, Yang Huang, Qianqian Chen, Chun Lin

**Affiliations:** 1 Fujian Medical University, Basic Medical College, Laboratory of Pain Research, Key Laboratory of Brain Aging and Neurodegenerative Diseases, Neuroscience Research Center, Fuzhou City, Fujian Province 350108, PR China; 2 Department of Pathology, Pingxiang People's Hospital, Pingxiang 337000, Jiangxi, PR China; University of Texas Medical Branch, UNITED STATES

## Abstract

**Background:**

The pathogenesis of multiple chronic visceral pain syndromes, such as irritable bowel syndrome (IBS), is not well known, and as a result current therapies are ineffective. The objective of this study was to investigate the effect of spinal protein kinase M zeta (PKMζ) on visceral pain sensitivity in rats with IBS to better understand the pathogenesis and investigate the effect of zeta inhibitory peptide (ZIP) as a therapy for chronic visceral pain.

**Methods:**

Visceral hypersensitivity rats were produced by neonatal maternal separation (NMS). Visceral pain sensitivity was assessed by electromyographic (EMG) responses of abdominal muscles to colorectal distention (CRD). Spinal PKMζ and phosphorylated PKMζ (p-PKMζ) were detected by western blot. Varying doses of ZIP were intrathecally administered to investigate the role of spinal PKMζ in chronic visceral hypersensitivity. The open field test was used to determine if ZIP therapy causes spontaneous motor activity side effects.

**Results:**

Graded CRD pressure significantly increased EMG responses in NMS rats compared to control rats (*p* < 0.05). p-PKMζ expression increased in the thoracolumbar and lumbosacral spinal cord in the IBS-like rats with notable concomitant chronic visceral pain compared to control rats (*p* < 0.05). EMG data revealed that intrathecal ZIP injection (1, 5, and 10 μg) dose-dependently attenuated visceral pain hypersensitivity in IBS-like rats.

**Conclusions:**

Phosphorylated PKMζ may be involved in the spinal central sensitization of chronic visceral hypersensitivity in IBS, and administration of ZIP could effectively treat chronic visceral pain with good outcomes in rat models.

## Introduction

Patients with irritable bowel syndrome (IBS) suffer from chronic visceral pain, which markedly affects patient quality of life and causes socioeconomic burden [1]. There is no satisfactory treatment for IBS at present because its underlying pathophysiology is poorly understood [2]. Thus, the search for effective IBS therapeutic strategies remains a significant clinical challenge.

Pain maintenance is associated with long-term potentiation (LTP). Protein kinase M zeta (PKMζ) is an atypical specific protein kinase C (PKC) that has sustained activation due to a lack of an auto-inhibitory regulatory domain. A previous study demonstrated that PKMζ is required for LTP [3, 4]. Recent research demonstrated that PKMζ provided a unified mechanism for long-term functional and structural modifications of synapses [5]. PKMζ is involved in persistent pain associated with inflammation and neuropathic pain [6]. Myristoylated zeta inhibitory peptide (ZIP) is a specific inhibitor of PKMζ that targets the auto-inhibitory pseudosubstrate fragment of PKC. ZIP binds an acidic surface on the Phox and Bem1 (PB1) domain of p62, an interaction validated by peptide array analysis. Results showed that ZIP abolished nociceptive sensitization caused by inflammation [7].

However, it remains unclear whether spinal PKMζ is involved in chronic visceral hyperalgesia in IBS. Furthermore, there is no evidence that ZIP inhibits chronic visceral hypersensitivity in IBS. The objective of this study was to investigate the effect of spinal PKMζ on visceral hypersensitivity in rats with IBS to better understand its pathogenesis. We also sought to investigate the effect of ZIP as a therapy for chronic visceral pain.

## Materials and Methods

### Experimental Animals

All neonatal male Sprague Dawley rats (3 days old) were obtained from the Laboratory Animal Center of Fujian Medical University (Animal approval number: SCXK 2012–0001). On postnatal day (PND) 22 neonates were separated from their mothers. Six young rats were placed in a little space with sawdust bedding, supplied food and water freely, and maintained on a 12-hours light/dark cycle. All procedures were conducted during the light cycle. Experiments were performed when the rats were 8 weeks old. Food and water were supplied ad libitum to all rats. The rats were monitored routinely at least once daily during the experimental procedures. Occasionally, the individual weakest neonates died due to insufficient nursing. Generally, this happened if a litter was too large for one nest and the mother could not adequately nurse all of the neonates. If the animals became severely ill prior to experimental endpoints, they were euthanized by an intraperitoneal injection of a lethal dose of pentobarbital sodium. The clinical signs of illness included sustained weight loss, self-destructive behavior, abnormal reaction of the central nervous system, and any obvious functional injury. Rats were gently handled before experiments to alleviate stress and anesthetized during recording. All animal procedures were approved by the Committee for Care and Use of Laboratory Animals at Fujian Medical University.

### Rat model of visceral hypersensitivity

A previous study reported that repeated neonatal maternal separation (NMS) could induce visceral hypersensitivity in adult rats, mimicking the main pathophysiological characteristics of IBS in humans [8]. Coutinho and O'Mahony *et*. *al*., reported that 180 minutes of daily maternal separation from PND 2–14 could induce visceral hypersensitivity [8, 9]. Based on this protocol, we initiated NMS by separating neonates from their mothers and placing them in new cages for 180 minutes and then returning the neonates to their mothers daily from PND 3–21 [10]. Control neonates remained with their mothers until PND 22.

### Assessment of visceral hypersensitivity by electromyographic (EMG) recordings

Visceral pain was measured by EMG recordings in 8 week old rats [11–13]. Rats were maintained under light isoflurane anesthesia using anesthetic equipment (VMR, Matrx, USA). Two silver electrodes were inserted into the abdominal muscles to measure EMG induced by graded colorectal distension (CRD). A balloon lubricated with glycerol was inserted into the colorectum. After rapid inflation, the balloon pressure was increased to 20, 40, 60, and 80 mmHg. The colorectum was distended by gradual balloon dilatation for 10 seconds under each pressure followed by a 4 minute resting period. EMG responses were recorded by a RM6240BD system (Chengdu, China). The EMG magnitude under different CRD was recorded three times. An average of three recordings was used for each CRD. EMG data were derived by subtracting the mean baseline amplitude (10 seconds pre-distention period) from the mean EMG amplitude of each pressure.

### Intrathecal catheterization and drug administration

Rats were anesthetized with barbanylum (8%, 0.1 ml/ 100 g). A sterile polyethylene catheter (PE10 tubing, Becton Dickinson) was introduced at the lumbar (L)6/ sacral (S)1 interspace and threaded into the lumbar enlargement. Rats recovered for 1 week after intrathecal cannulation. Various doses (1, 5, and 10 μg) of ZIP diluted in sterile 0.9% saline were administered in a volume of 10 μl followed by a 10 μl saline flush. Visceral hypersensitivity measurements were recorded 30 minutes before and after intrathecal administration. The EMG amplitude was recorded every 30 minutes for 180 minutes after intrathecal injection of 10 μg ZIP in IBS-like rats to observe the time course of ZIP inhibition.

### Western blotting

Expression of spinal PKMζ and phosphorylated PKMζ (p-PKMζ) were detected by western blot in control and IBS-like rats. Equal amounts of protein (30 μg) from the spinal thoracolumbar and lumbosacral segments of rats were separated by electrophoresis, then transferred from gels to PVDF membranes (Invitrogen, USA). The membranes were probed with the following antibodies: rabbit anti-PKMζ monoclonal antibody (1:500, Santa Cruz Biotechnology Inc, USA), rabbit anti-p-PKMζ monoclonal antibody (1:400, Santa Cruz Biotechnology Inc, USA), and rabbit anti-GAPDH primary antibody (1:3,000, Bioworld Technology Inc, USA). The blots were washed and incubated with peroxidase-conjugated goat anti-rabbit IgG (1:1,000, Abcam, USA). The western blot data are presented as the optical density ratio of the detected protein relative to GAPDH.

### Open field test

Effects of intrathecal ZIP injection on spontaneous motor activity in IBS-like rats were assessed using the open field test [14]. Rats were placed in a gray box (100 cm x 100 cm x 60 cm) with a black bottom that was open at the top. Rats were gently placed in the center bottom of the box. The movement locus was video recorded for 5 minutes. Rats were tested at 30 minutes after intrathecal ZIP injection. Total walking distance (m) and average speed (cm/s) were measured. The box was thoroughly cleaned to remove odor cues after every test.

### Statistical analysis

All data are presented as mean ± standard error of the mean (SEM). A two tailed independent sample t-test was used to determine difference between controls and IBS-like rats in the EMG response to CRD. Results of western blot and open field tests were analyzed by a two tailed independent sample t-test. The paired t-test was used to compare the EMG response to CRD pre and post injection. Data analysis was performed using SPSS 11.7. A *p* value < 0.05 was considered statistically significant.

## Results

### NMS causes chronic visceral hypersensitivity in adult rats

EMG recordings were used to assess visceral sensitivity in rats at 8 weeks of age. Graded CRD pressure significantly increased EMG responses in NMS rats compared to control rats. In NMS rats, the EMG amplitude increased compared to the control group by 102.9% under 20 mmHg CRD, 324.98% under 40 mmHg CRD, 493.65% under 60 mmHg CRD, and 506.57% under 80 mmHg CRD (two tailed independent sample t-test, *p* < 0.05; Table 1). These results suggest that NMS remarkably increases visceral sensitivity in adult rats compared to controls.

**Table 1:** EMG responses to CRD in control and IBS-like rats (n = 10).

	EMG amplitude (% from baseline, $\bar{\chi }$± SEM)			
	20mmHg	40mmHg	60mmHg	80mmHg
Control	12.35± 2.77	203.17±26.68	321.45±21.77	393.84±28.47
IBS	115.25±33.72^*^	528.15±67.13^*^	815.10±60.29^*^	900.41±94.41^*^

*p < 0.05 *vs*. Control rats. EMG: electromyography; CRD: colorectal distention.

### p-PKMζ is increased in the thoracolumbar spinal cord in IBS-like rats

**Fig 1:**
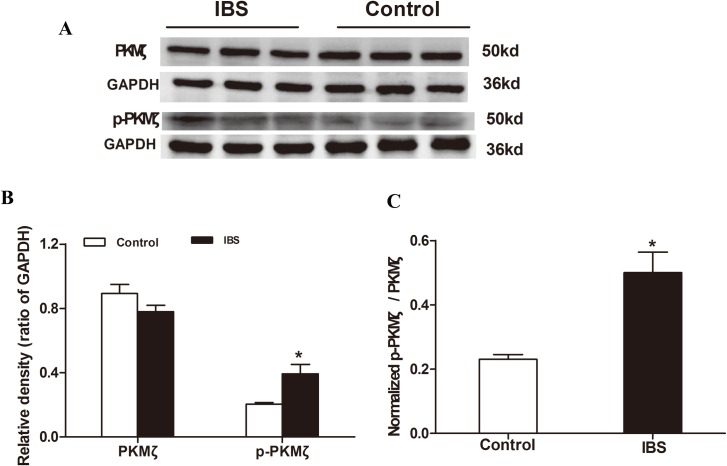
PKMζ expression in the thoracolumbar spinal cord in rats. A: Western blot results show the expression of thoracolumbar spinal cord PKMζ and p-PKMζ in rats. B: Expression of p-PKMζ was significantly increased in IBS-like rats; n = 3 for each group. C: The ratio of normalized p-PKMζ to normalized PKMζ in the thoracolumbar spinal cord of rats. The ratio was enhanced in IBS-like rats. *p < 0.05 *vs*. Controls. IBS: irritable bowel syndrome.

Western blot results demonstrated that p-PKMζ expression in the thoracolumbar spinal cord in IBS-like rats was significantly increased by 95% (from 0.20 ± 0.01 to 0.39 ± 0.06) compared to control rats (two tailed independent sample t-test, *p* < 0.05; Fig 1A and 1B). However, PKMζ expression in the thoracolumbar spinal cord (0.78 ± 0.04) was not significantly changed in IBS-like rats compared to controls (0.89 ± 0.06) (two tailed independent sample t-test, *p* > 0.05; Fig 1A and 1B). Furthermore, the ratio of normalized p-PKMζ to PKMζ in IBS-like rats was significantly enhanced by 117% (from 0.23 ± 0.01 to 0.50 ± 0.06) (*p* < 0.05; Fig 1C). GAPDH was used as an internal control for normalization of western blots.

### p-PKMζ is increased in the lumbosacral spinal cord in IBS-like rats

**Fig 2:**
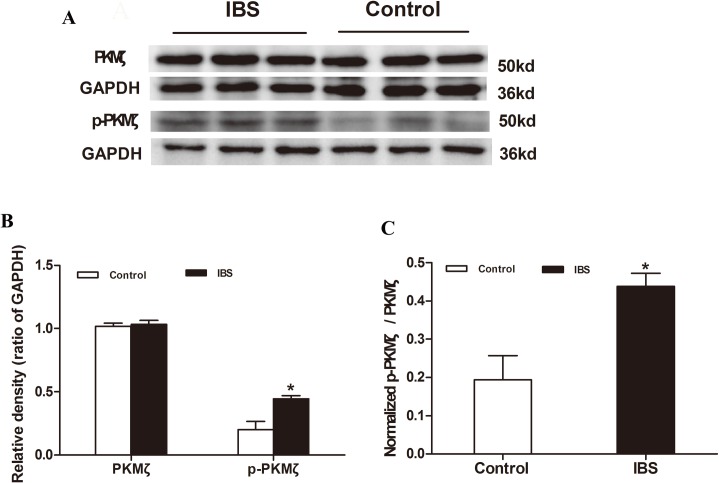
PKMζ expression in the lumbosacral spinal cord in rats. A: Western blot results show the expression of lumbosacral spinal cord PKMζ and p-PKMζ in rats. B: Expression of p-PKMζ was significantly increased in IBS-like rats; n = 3 for each group. C: The ratio of normalized p-PKMζ to normalized PKMζ in the thoracolumbar spinal cord of rats. The ratio was enhanced in IBS-like rats. *p < 0.05 *vs*. Controls. IBS: irritable bowel syndrome.

Western blot results demonstrated that p-PKMζ expression in the lumbosacral spinal cord in IBS-like rats was significantly increased by 120% (from 0.20 ± 0.06 to 0.44 ± 0.02) compared to controls (two tailed independent sample t-test, *p* < 0.05; Fig 2A and 2B). However, PKMζ expression in the lumbosacral spinal cord (1.03 ± 0.03) was not significantly changed in IBS-like rats compared to controls (1.02 ± 0.02) (two tailed independent sample t-test, *p* > 0.05; Fig 2A and 2B). Furthermore, the ratio of normalized p-PKMζ to PKMζ in IBS-like rats was significantly enhanced by 117% (from 0.19 ± 0.06 to 0.43 ± 0.04) (*p* < 0.05; Fig 2C). GAPDH was used as an internal control for normalization of western blots.

### ZIP alleviates visceral hypersensitivity in IBS-like rats

**Fig 3:**
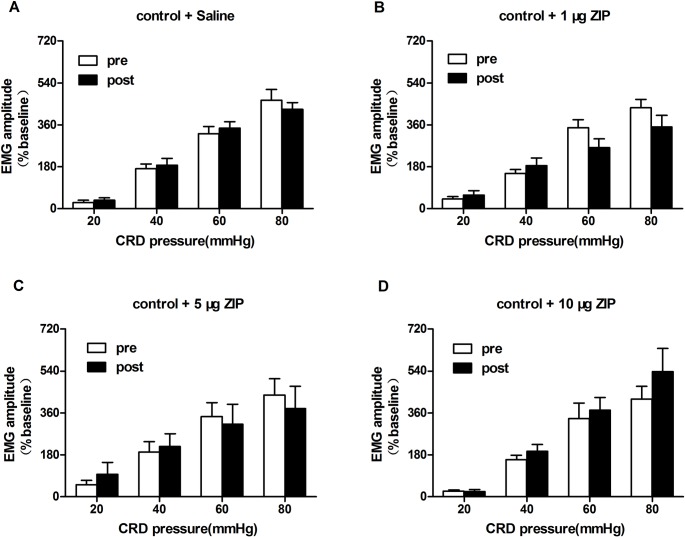
Effects of intrathecal ZIP injection on EMG response in control rats. A: EMG amplitude at 20–80 mmHg CRD pressure after intrathecal saline in controls. B: EMG amplitude at 20–80 mmHg CRD pressure after 1 μg ZIP in controls. C: EMG amplitude at 20–80 mmHg CRD pressure after 5 μg ZIP in controls. D: EMG amplitude at 20–80 mmHg CRD pressure after 10 μg ZIP in controls. ZIP had no effect on chronic visceral hypersensitivity in controls; n = 10, respectively. EMG: electromyography; CRD: colorectal distention; IBS: irritable bowel syndrome; ZIP: PKMζ inhibitor.

**Fig 4:**
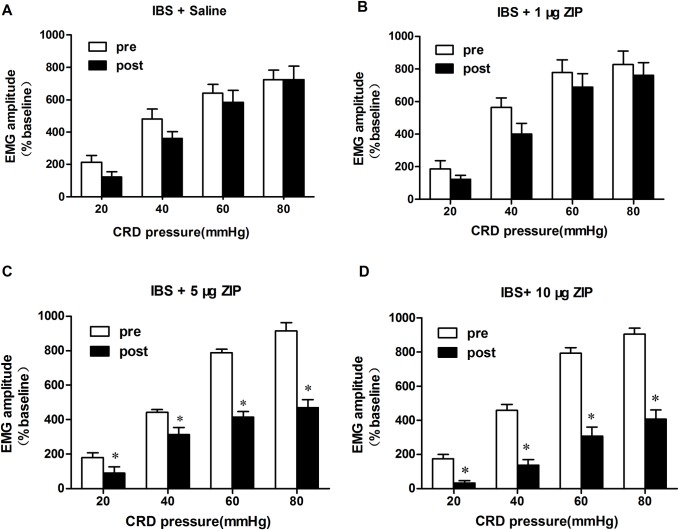
Inhibitory effect of intrathecal ZIP injection on EMG response in IBS-like rats. A: EMG amplitude at 20–80 mmHg CRD pressure after saline in IBS-like rats. B: EMG amplitude at 20–80 mmHg CRD pressure after 1 μg ZIP in IBS-like rats. C: EMG amplitude at 20–80 mmHg CRD pressure after 5 μg ZIP in IBS-like rats. D: EMG amplitude at 20–80 mmHg CRD pressure after 10 μg ZIP in IBS-like rats. ZIP dose-dependently inhibited the chronic visceral hypersensitivity in IBS-like rats; n = 10, respectively. *p < 0.05 *vs*. pre-drug. EMG: electromyography; CRD: colorectal distention; IBS: irritable bowel syndrome; ZIP: PKMζ inhibitor.

Our results showed that saline and varying doses of ZIP (1, 5, and 10 μg) injected *via* spinal administration did not affect EMG in control rats (paired t-test, *p* > 0.05; Fig 3A–3D). We observed no significant changes in EMG responses to 20–80 mmHg CRD after saline and the minimum dose of ZIP (1 μg) compared to the baseline EMG in IBS-like rats (*p* > 0.05; Fig 4A and 4B). After spinal injection of 5 μg ZIP, the EMG amplitude was significantly decreased by 50%, 29%, 47%, and 49% under 20, 40, 60, and 80 mmHg CRD, respectively, compared to EMG responses in IBS-like rats before ZIP injection (*p* < 0.05; Fig 4C). After spinal injection of 10 μg ZIP, the EMG amplitude was significantly decreased by 82%, 70%, 61%, and 55% under 20, 40, 60, and 80 mmHg CRD, respectively, compared to EMG responses in IBS-like rats before ZIP injection (*p* < 0.05; Fig 4D). These results show that ZIP dose-dependently inhibits chronic visceral hypersensitivity.

### Time course of ZIP suppression efficiency in chronic visceral hypersensitivity in IBS-like rats

**Fig 5:**
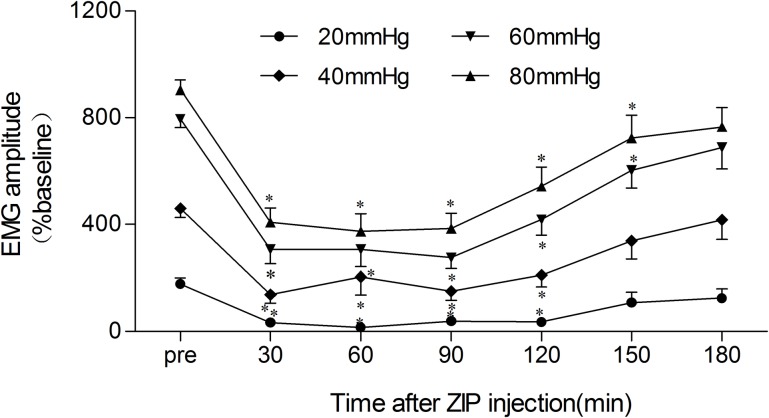
The duration curve of the inhibitory effect of intrathecal ZIP injection on visceral hypersensitivity in IBS-like rats. Duration curve of the inhibitory effect of 10 μg ZIP on EMG at 20–80 mmHg of CRD in IBS-like rats. The maximal suppression was recorded at 30 minutes after ZIP injection in IBS-like rats. The EMGs under 20–80 mmHg CRD were significantly decreased at 30–90 minutes after 10 μg ZIP injection; n = 10, respectively. *p < 0.05 *vs*. pre-drug. EMG: electromyography; IBS: irritable bowel syndrome; CRD: colorectal distention; ZIP: PKMζ inhibitor.

We recorded the EMG amplitude in response to 20–80 mmHg CRD pressure every 30 minutes for 180 minutes after intrathecal injection of 10 μg ZIP in IBS-like rats to observe the time course of ZIP inhibition. The EMG amplitude decreased to the lowest level 30 to 90 minutes after ZIP injection under 20, 40, 60, and 80 mmHg CRD, and then gradually increased and nearly returned to normal levels at 180 minutes after ZIP injection. These results (Fig 5) show that the maximal inhibitory effect occurs at 30 minutes and is maintained for about 120 minutes after ZIP injection.

### ZIP does not affect motor function in IBS-like or control rats

Neither the highest dose of ZIP (10 μg) nor control saline affected total walking distance and average speed in IBS-like and control rats, assessed at 60 minutes post-injection in the open field test (two tailed independent sample t-test, *p* > 0.05; Table 2). This indicates that intrathecal injection of ZIP has no effect on spontaneous motor activity in rats.

**Table 2:** Effect of intrathecal ZIP injection on open field test in rats.

	Total walking distance (m)		Average speed (cm/s) ($\bar{\chi }$ ± SEM)	
	Saline	ZIP	Saline	ZIP
Control	18.18±2.55	17.57±3.30	6.06±0.80	5.86±1.10
IBS	9.73±0.70	11.71±2.59	3.25±0.23	3.91±0.86

IBS: irritable bowel syndrome; ZIP: PKMζ inhibitor.

## Discussion

The current study demonstrates that PKMζ is necessary and sufficient for visceral hypersensitivity [3, 4, 15]. PKMζ is necessary for storage of spatial information and involved in persistent spinal cord nociception, specifically after chronic pain caused by peripheral inflammation [3, 16]. Previous results from our laboratory demonstrated that hippocampal PKMζ contributed to the maintenance of visceral hypersensitivity in IBS-like rats [10]. Currently, we show that PKMζ, especially p-PKMζ, plays an important role in spinal hyperalgesia in IBS-like rats. First, p-PKMζ expression was increased in the spinal cord in IBS-like rats compared to controls. Secondly, ZIP, the inhibitor of PKMζ, significantly attenuated visceral hypersensitivity in a dose-dependent manner in IBS-like rats compared to control rats. Finally, the inhibitory effect of ZIP on chronic visceral hypersensitivity lasted for a few hours.

### Spinal PKMζ plays a role in chronic visceral hypersensitivity in IBS-like rats

PKMζ, an atypical protein kinase C, has an independent catalytic domain but no regulatory domain; thus, PKMζ lacks auto-inhibition from the regulatory domain and is therefore persistently active. This kind of activity is relatively low, but it can be greatly enhanced after phosphorylation [17, 18]. PKMζ is likely the key effector molecule for maintaining synaptic plasticity [19]. There is growing evidence that PKMζ is involved in persistent nociceptive processing in the central nervous system. p-PKMζ was highly expressed in all categories of dorsal root ganglion (DRG) neurons in models of inflammatory pain [20, 21]. PKMζ in the anterior cingulate cortex (ACC) contributed to pain maintenance induced by experimental tooth movement [22]. Surgical injuries following peripheral inflammation priming substantially increased p-PKMζ expression in the spinal cord. Expression of PKMζ and p-PKMζ in the ACC were both significantly increased in neuropathic pain, suggesting that PKMζ is important for maintaining synaptic plasticity in the ACC [15]. Our previous study demonstrated that hippocampal p-PKMζ was significantly increased in IBS-like rats [10]. Our present research indicates that p-PKMζ expression in the spinal cord increases in IBS-like rats, which is consistent with our previous work. It is reasonable to draw a conclusion that spinal PKMζ contributes to the central sensitization of chronic visceral hypersensitivity.

However, research also showed that expression of PKMζ and p-PKMζ in the hippocampus and spinal cord was not significantly changed after nerve injury. Furthermore, neuropathic allodynia was not subdued by intrathecal ZIP injection after peripheral nerve injury [15]. Complete Freund’s Adjuvant (CFA)-induced mechanical and thermal pain was alleviated by ZIP. However, this effect could not inhibit mechanical allodynia and hyperalgesia in neuropathic pain [21]. Our present study indicates that spinal PKMζ contributes to the maintenance of nociceptive plasticity in chronic visceral hypersensitivity. Thus, there are likely different modes of pathogenesis of nociceptive plasticity underlying these types of pains. In our study, the effects of ZIP varied in neuropathic pain and chronic visceral pain, suggesting that changes in PKMζ vary in different regions of the central nervous system, and there are probably some molecular mechanistic differences of nociceptive plasticity between neuropathic pain and chronic visceral pain. Our previous research indicated that hippocampal PKMζ contributed to the maintenance of LTP in IBS-like rats [10]. In future studies we will investigate whether PKMζ contributes to the pathogenesis of chronic visceral hypersensitivity in other regions of the central nervous system, such as the ACC.

In rats the descending colon and rectal visceral nerve fibers project into the L6/S2 segment of the spinal cord through the pelvic nerve (parasympathetic) and into the T13/L2 segment through the hypogastric nerve (sympathetic) [22–25]. We previously observed that numbers of activated afferent nerve fibers increased significantly in the thoracolumbar and lumbosacral segment of the spinal cord after CRD in IBS-like rats [13, 26], which suggests that these two regions participate in the central sensitization of chronic visceral hypersensitivity. In our study, we chose the thoracolumbar and lumbosacral segment of the spinal cord to measure expression levels of PKMζ and p-PKMζ. Our current findings support our previous research.

### ZIP attenuates visceral hypersensitivity in a dose-dependent manner in IBS-like rats

ZIP is a PKMζ inhibitor [27]. Previous results showed that incision sensitization was significantly inhibited after intrathecal ZIP injection following carrageenan-induced priming in a peripheral inflammation pain model [20]. After injection of ZIP, sensitization to both hindpaw injection of prostaglandin E2 and intrathecal administration of metabotropic glutamate receptor 1 and 5 agonist after the resolution of interleukin-6 (IL-6)-induced allodynia was completely abolished [7]. These results indicate that PKMζ is involved in inflammation-primed persistent nociceptive sensitization *via* a ZIP-reversible mechanism. In addition, injection of ZIP into the ACC blocked nociceptive sensitization after peripheral nerve injury [15]. Other evidence demonstrated that ZIP injected into the rACC reversed spinal nerve ligation-induced spontaneous pain [28]. These studies show that ZIP can reverse the nociceptive sensitization of neuropathic pain in the ACC. Hence, PKMζ contributes to the maintenance of somatalgia and ZIP can inhibit this effect. Some evidence showed that ZIP inhibited hypersensitivity even when priming induced allodynia disappeared [7, 29]. Our present study revealed that after intrathecal microinjection of ZIP, visceral hypersensitivity significantly decreased in a dose-dependent manner in the IBS-like rats, but ZIP had no effect in the control rats. Thus, PKMζ is involved in the central sensitization of chronic visceral hypersensitivity *via* a ZIP-reversible mechanism.

In the inflammation model of pain, increased spinal PKMζ expression was sustained, but expression of other atypical PKCs (PKCζ and PKCι/l) only transiently increased. Furthermore, ZIP reduced allodynia sensitization of spinal cord dorsal horn neurons. However, post-treatment with NPC-15437 (inhibitor of all PKC isoforms except PKMζ) did not alleviate persistent nociception or allodynia caused by cutaneous injury or spinal stimulation. Therefore, PKMζ may be the critical factor for maintaining spinal plasticity in persistent pain, while the other atypical PKCs may contribute to persistent nociception. It has recently been suggested that ZIP not only blocks PKMζ but also inhibits other atypical PKCs [30]. Thus, additional research is needed to determine whether other atypical PKCs participate in the pathogenesis of chronic visceral hypersensitivity. We will use NPC-15437 to further verify the function of PKMζ in chronic visceral hypersensitivity of IBS in future studies.

In this study we determined the effective window of ZIP. In IBS-like rats, visceral hypersensitivity decreased significantly 30–90 minutes after intrathecal ZIP injection, and then gradually increased and returned to baseline levels 3 hours later. The inhibitory effect of ZIP on the incision sensitization was long lasting; incision pain caused by carrageenan priming was alleviated for at least 16 days after ZIP injection [20]. ZIP completely abolished sensitization precipitated after injection of the hindpaw with prostaglandin E2 or dihydroxyphenylglycine in the interleukin-6-induced allodynia, and the effect lasted for several days, but it could not alter the IL-6-induced initial allodynia [7]. Tooth pain caused by tooth movement was alleviated after the injection of ZIP into the ACC. The inhibitory effect lasted for at least 1 day [22]. In both pain models, allodynia in the late chronic post-ischemia pain (3 weeks post-injury, which depends on central changes) was significantly attenuated by ZIP, but allodynia in the chronic constriction injury of the sciatic nerve (3–4 days post-injury, which depends of ongoing peripheral inputs) was not significantly inhibited by ZIP [6]. Our study demonstrates that ZIP significantly attenuates adult persistent visceral chronic hypersensitivity caused by NMS, but with a shorter therapeutic window compared to the above mentioned research. We suggest that this difference is related to the different action modes and doses of ZIP on various kinds of pain.

### Spinal PKMζ may be a therapeutic target for the treatment of chronic visceral hypersensitivity in IBS

There is no satisfactory treatment of IBS at present [2, 31]. Very few drugs have been specifically approved to treat visceral pain syndromes. Zelnorm, a partial serotonin receptor 4 agonist, was used for the short-term treatment of women with IBS; however, it was subsequently withdrawn because of serious side effects [32]. N-methyl-D-aspartic acid receptor inhibitors have also been used to treat IBS, but these also cause numerous side effects, such as memory impairment, psychotomimetic effects, ataxia, and motor incoordination [33]. Acupuncture has been proposed as another possible treatment for IBS, but there is currently insufficient evidence that this is an effective treatment [34–37]. Studies have shown that injection of ZIP into the ACC after peripheral nerve injury erased synaptic potentiation and blocked behavioral sensitization [15]. Although our study demonstrates the acute effects of ZIP, we presume that higher doses or repeated administrations may have long-term effects. If this is correct there will be significant potential for developing therapies for chronic visceral pain.

## Conclusion

According to our research, spinal PKMζ is involved in central sensitization in chronic visceral hypersensitivity. ZIP can significantly attenuate persistent visceral chronic hypersensitivity. Importantly, ZIP only influences sensory functions, but has no effect on motor function in rats. It is reasonable to suggest that PKMζ may be developed as a new therapeutic target to prevent chronic visceral hypersensitivity in IBS, and ZIP may be a selectable therapeutic drug for patients with IBS.
